# Toward atomic force microscopy and mass spectrometry to visualize and identify lipid rafts in plasmodesmata

**DOI:** 10.3389/fpls.2014.00234

**Published:** 2014-05-30

**Authors:** Pamela A. Naulin, Natalia A. Alveal, Nelson P. Barrera

**Affiliations:** Department of Physiology, Faculty of Biological Sciences, Pontificia Universidad Católica de ChileSantiago, Chile

**Keywords:** mass spectrometry (MS), atomic force microscopy (AFM), lipid raft, membrane proteins, plasmodesmata (PDs)

## Abstract

Plant cell-to-cell communication is mediated by nanopores called plasmodesmata (PDs) which are complex structures comprising plasma membrane (PM), highly packed endoplasmic reticulum and numerous membrane proteins. Although recent advances on proteomics have led to insights into mechanisms of transport, there is still an inadequate characterization of the lipidic composition of the PM where membrane proteins are inserted. It has been postulated that PDs could be formed by lipid rafts, however no structural evidence has shown to visualize and analyse their lipid components. In this perspective article, we discuss proposed experiments to characterize lipid rafts and proteins in the PDs. By using atomic force microscopy (AFM) and mass spectrometry (MS) of purified PD vesicles it is possible to determine the presence of lipid rafts, specific bound proteins and the lipidomic profile of the PD under physiological conditions and after changing transport permeability. In addition, MS can determine the stoichiometry of intact membrane proteins inserted in lipid rafts. This will give novel insights into the role of membrane proteins and lipid rafts on the PD structure.

## Introduction

Plamodesmata (PDs) are nanopores connecting the cytoplasm of adjacent cells to facilitate intercellular communication. PDs generate symplastic communication pathways to transport small molecules below the size exclusion limit and selected bigger molecules. This in turn plays important roles for the cell fate and development, viral movement and transport of metabolites and miRNA (Bouyer et al., 2008; Lucas et al., 2009; Carlsbecker et al., 2010; Miyashima et al., 2011; Furuta et al., 2012)

PDs are structurally composed by a continuous plasma membrane (PM) between two adjacent cells with an axial highly packed central element of endoplasmic reticulum named desmotubule (Hepler, 1982; Tilney et al., 1991; Ding et al., 1992). The cell wall surrounding the channel is rich in pectin and depositions of β-1,3-glucan (callose) in the plasmodesma (PD) neck zone. To date there is consensus that these depositions regulate the molecular size of the transported molecules through PD (Roy et al., 1997; Botha and Cross, 2000; Levy et al., 2007; Guseman et al., 2010). In recent years, there have been considerable efforts carried out to determine the molecular composition of the PD. In particular, using nano-LC ion trap MS/MS, Fernandez-Calvino et al. (2011) have done a proteomic analysis of PD vesicles and identified 1341 proteins that putatively belong to the PD (five of them were confirmed to be located inside the PD by confocal microscopy experiments), including glycosylphosphatidyl inositol (GPI)-anchored proteins. This family of proteins are anchored to the membrane and present high affinity for sterol containing lipid bilayers, which suggest that PD membrane could be forming lipid rafts (Mongrand et al., 2010; Salmon and Bayer, 2012). Membrane lipid rafts are defined as dynamical assemblies of sphingolipids and sterols (Lingwood and Simons, 2010). Supporting this hypothesis, Remorin protein, a lipid raft marker, was accumulated in the PD (Raffaele et al., 2009). Nevertheless, there is no experimental evidence showing the presence of lipid rafts in PDs, neither their lipid composition nor the location of intact specific membrane proteins. In this perspective article we propose a series of experimental approaches to get insights into these important issues by using atomic force microscopy (AFM) and mass spectrometry (MS).

## Atomic force microscopy imaging

AFM was invented (Binnig et al., 1986) to analyse only conductive samples and since then has advanced enormously specially with the design of new methodologies to study biological samples such as proteins, DNA and lipid bilayers under physiological conditions (Muller, 2008; Shahin and Barrera, 2008; Picas et al., 2012; Whited and Park, 2013). Basically AFM consists of a sharp tip around 10 nm radius attached to a flexible cantilever scanning over a sample to reconstruct its three dimensional topography. Because of the tip width further geometric deconvolution is needed to improve the sample lateral resolution (x, y plane) until nanometric dimension. On the other hand, the sample height (z plane) can achieve sub angstrom resolution based on finely tuning interaction force between the tip and sample. Depending upon samples properties, scanning can be done continuously (contact mode) or intermittently (tapping mode) for hard or soft samples respectively (for more details see Shahin and Barrera, 2008).

## AFM and biological membranes

AFM has long been used to visualize lipid bilayers with a height resolution near to 0.1 nm (Mou et al., 1995; Dufrene et al., 1997; Grandbois et al., 1998; Hollars and Dunn, 1998; Rinia et al., 1999; McKiernan et al., 2000; Reviakine et al., 2000; Muresan and Lee, 2001) which has allowed dynamical detection of microdomains (rafts) in lipid bilayers and in native membranes *in vitro* (Dufrene et al., 1997; Giocondi et al., 2000, 2001, 2004; Yuan et al., 2002; Lawrence et al., 2003; Anderton et al., 2011). For example, using AFM (Lawrence et al., 2003) have studied in real time the effects of manipulating cholesterol levels in supported model membranes containing dioleoylphosphatidylcholine (DOPC) and sphingomyelin (SM). In absence of cholesterol, these membranes form small SM domains, which increase after cholesterol addition. An ordered and unique lipid raft domain is present at very high cholesterol concentrations, effect that is reversed once methyl-ß-cyclodextrin (MßCD, cholesterol chelator) is applied. In addition, time-lapse AFM has been used to visualize dynamical processes in living cells, like extension and retraction of lamellipodium in MCF-7 cells (Li et al., 2013). Recent AFM studies on native membranes have proved the presence of lipid rafts in erythrocytes with a size of 100–300 nm and irregular shape and height of 2–4 nm above membrane bilayer (Cai et al., 2012). Orsini et al. (2012) have shown detergent-resistant membranes (DRMs) in human breast cancer cells with sizes of 100–500 nm and heights 1–2 nm above the PM. Furthermore, they demonstrated the presence of flotillin-1, a specific raft marker. Altogether, these evidence highlight the usefulness of the AFM technique in the lipid rafts analysis.

## Mass spectrometry of membrane proteins and lipid rafts

MS determines both abundance and precise mass of biomolecules based on their ionization and mass/charge relationship in the gas phase (Barrera and Robinson, 2011). MS has emerged as a powerful tool to quantitatively analyse complex phospholipids such as those contained in lipid rafts, including glycerophospholipids and sphingolipids, from crude extracts (Pulfer and Murphy, 2003; Han and Gross, 2005). Interestingly, apart from proteomics information, MS has identified intact membrane proteins (Barrera et al., 2013), and also the stoichiometry and nature of lipids bound to them (Barrera et al., 2008, 2009). Altogether these data have shown that MS can provide structural aspects all the way through proteomics and lipidomics to stoichiometries of intact complexes.

A decade ago, a proteomic study identified 238 PM proteins from *Arabidopsis thaliana* (Alexandersson et al., 2004). They found 114 integral/GPI and 124 peripheral proteins; however only 180 out of the total proteins detected were classified as having a known function.

Lipid rafts in plants were suggested by the presence of a Triton X-100 insoluble PM fraction or DRM in tobacco cells. This fraction exhibited a different protein composition to that of PM, including GPI-anchored proteins (Peskan et al., 2000). Other proteins associated to DRMs comprise receptor-like kinases (RLKs), G-proteins (Morel et al., 2004), redox system proteins (Lefebvre et al., 2007) and stress associated proteins (Cacas et al., 2012). A proteomic strategy was developed to characterize membrane proteins associated to sterol containing DRMs fractions in *A. thaliana* (Kierszniowska et al., 2009). They found a considerable number of GPI-anchored proteins and other proteins with unknown function. Remorin protein, a molecular marker for lipid rafts in plants, has also been localized in the PD in *Solanaceae* family (Raffaele et al., 2009) In agreement to this, Fernandez-Calvino et al. (2011), via proteomics of the PD in *A. thaliana*, reported a variety of GPI-anchored proteins and remorin. Altogether, these data suggest that lipid rafts may constitute the PD.

Apart from lipid rafts, other cell membrane domains are tetraspanin-enriched microdomains (TEMs) (Hemler, 2005). Tetraspanins are integral transmembrane proteins which contain four transmembrane domains and two extracellular loops. Tetraspanins associates with cholesterol through a palmitate (S-acylation of the protein), and with gangliosides (Berditchevski, 2001; Boucheix and Rubinstein, 2001; Ono et al., 2001; Hemler, 2003, 2005). Most of their functions are involved in cell adhesion (to the extracellular matrix, other cells and pathogens), intercellular communication, membrane fusion and intracellular signaling. TEMs might enhance these processes by clustering functionally related molecules or by tightly packing a critical number of specific receptors at the PM (Yáñez-Mó et al., 2009). Lipid rafts and TEMs have similarities such as cholesterol enrichment (Le Naour et al., 2006) and localization in DRMs (Charrin et al., 2003). In contrast to lipid rafts, TEMs are mostly soluble in stronger non-ionic detergents, and resistant to cholesterol depletion (Claas et al., 2001), although partial disruption may be occasionally observed (Charrin et al., 2003). GPI-anchored proteins have not been detected in TEMs (Hemler, 2005). Based on tetraspanin identification and the absence of significant amounts of PM or endoplasmic reticulum markers in the PDs (Fernandez-Calvino et al., 2011), these nanopores could be constituted by highly specialized membrane microdomains that may contain TEMs. Indeed, it has been demonstrated the coalescence of lipid rafts and TEMs in human immunodeficiency virus type 1 (HIV-1) assembly sites on the PM by Förster resonance energy transfer (FRET) assay in living cells (Hogue et al., 2011). Furthermore, integrin-tetraspanin signaling complexes are partitioned into specific microdomains proximal to cholesterol-rich lipid rafts (Berditchevski, 2001). Table 1 shows a summary of PD associated proteins with structural properties identified or hypothesized.

**Table 1:** **Proteins localized in the PD**.

**Protein/Organism**	**Method**	**Mass (Da)/AGI code**	**X-ray structure homolog**	**Probable stoichiometry**	**Function**	**References**
Myosin VIII-A *A.thaliana*	Immunolocalization	130007.5/At1g50360	–	Dimer	ATP binding, motor activity	Reichelt et al., 1999; Golomb et al., 2008
Calreticulin *Maize*	Immunolocalization	48527/At1g56340	3O0V, 3O0W, 3O0X Kozlov et al., 2010; 3POS, 3POW Chouquet et al., 2011	Monomer	Chaperonin promoting folding, oligomeric assembly and quality control in the ER; interaction with TMV MP	Baluska et al., 1999; Chen et al., 2005
Pectin methyl esterase or PME *N. tabacum*	Immunolocalization	64148.6/At1g53840	–	–	Catalyze esterification of pectins; specifically binds to the TMV MP	Dorokhov et al., 1999; Chen et al., 2000
Class III peroxidase *L. esculentum*	Transmission-electron microscopy	39559.0/At1g71695	1SCH (Schuller et al., 1996)	–	Production de hydroxyl radicals	Ehlers and Van Bel, 2010
Beta-1,3-Glucanase (AtBG_ppap) *A. thaliana*	Proteomics/fluorescent protein fusion/confocal microscopy	45357.4/At5g42100	4GZI (Wojtkowiak et al., 2013)	Monomer	Degradation of callose; glycoside hydrolases; GPI-anchored PM protein	Bayer et al., 2006; Levy et al., 2007
Plasmodesmata Located Protein (PDLP) *A. thaliana*	Proteomics/fluorescent protein fusion/confocal microscopy	32606.6/At5g43980	–	–	Membrane receptor type 1	Bayer et al., 2006; Thomas et al., 2008
Plasmodesmal Callose Binding (PDCB) *A. thaliana*	Proteomics/fluorescent protein fusion/confocal microscopy	20364.4/At5g61130	–	–	GPI-anchored PM protein	Bayer et al., 2006; Simpson et al., 2009
LRR RLK *A. thaliana*	Proteomics/fluorescent protein fusion/confocal microscopy	114874/At1g56145	3BEL (Xu et al., 2008)	Homodimer	Signaling	Walker, 1994; Fernandez-Calvino et al., 2011
Tetraspanin (TET3) *A. thaliana*	Proteomics/fluorescent protein fusion/confocal microscopy	31887.9/At3g45600	1G8Q (Kitadokoro et al., 2001)	Homodimer- Heterodimer Boavida et al., 2013	Formation of membrane microdomains	Silvie et al., 2006; Espenel et al., 2008; Fernandez-Calvino et al., 2011
crRLK1L *A. thaliana*	Proteomics/fluorescent protein fusion/confocal microscopy	91822.4/At5g24010	3BEL (Xu et al., 2008)	Homodimer	Signaling	Walker, 1994; Fernandez-Calvino et al., 2011
S-domain RLK *A. thaliana*	Proteomics/fluorescent protein fusion/confocal microscopy	96464.9/At4g21380	3BEL (Xu et al., 2008)	Homodimer	Signaling	Walker, 1994; Fernandez-Calvino et al., 2011
Beta-1,6-N-acetylglucosaminyl transferase-like enzyme (AtGnTL) *A. thaliana*	Fluorescent fusion protein via confocal microscopy	39516.5/At3g52060	2GAM (Pak et al., 2006)	–	Glycosyltransferase	Zalepa-King and Citovsky, 2013
Remorin *S. lycopersicum*	Immunolocalization	20968/At2g45820	–	Homotrimer	–	Raffaele et al., 2009; Perraki et al., 2012
Actin *C. corallina*	Immunolocalization	41735.4/At5g09810	–	–	Cytoskeleton protein	Blackman and Overall, 1998
LYM2 *A. thaliana*	Proteomics/fluorescent protein fusion/confocal microscopy	37721,6/At2g17120	–	–	GPI-anchored PM protein; pattern-recognition receptor of pathogen	Fernandez-Calvino et al., 2011; Faulkner et al., 2013

*This table is not an exhaustive list of all proteins that have been associated with PD*.

*RLK, Receptor-Like Kinase; LRR RLK, Leucine Rich Repeat Receptor –Like Kinases; crRLK1L, Catharanthus roseus Receptor-Like kinase1-like; DUF, Domain of Unknown Function; TMV MP, movement protein encoded by tobacco mosaic virus; LYM2, Lysin motif domain-containing glycosylphosphatidylinositol-anchored protein 2*.

Mongrand et al. (2004) analyzed the lipidomics of DRMs isolated with Triton X-100 from tobacco PM. These microdomains mostly contained a sphyngolipid, named glucosylceramide (GluCer), and sterols such as stigmasterol, sitosterol, 24-methylcholesterol, and cholesterol. Using TLC and gas chromatography/mass spectrometry (GC/MS), two phosphoinositides PI(4)P y PI(4,5)P2 were quantified in DRMs of PM from tobacco and BY-2 cells (Furt et al., 2010). Both phosphoinositides represent less than 5% of total lipids in tobacco PM; however its relative amount is increased 11 times in membrane rafts. In addition, structural phospholipids, such as phosphatidylcholine, phosphatidylethanolamine, phosphatidylinositol, phosphatidylserine, phosphatidic acid were not abundant in DRMs compared to the PM (Mongrand et al., 2004). Although MS has advanced the knowledge of lipid composition in membrane rafts (Mongrand et al., 2010), its application on the study of PD is still absent.

## Characterization of the PD membrane via AFM and MS

**Figure 1:**
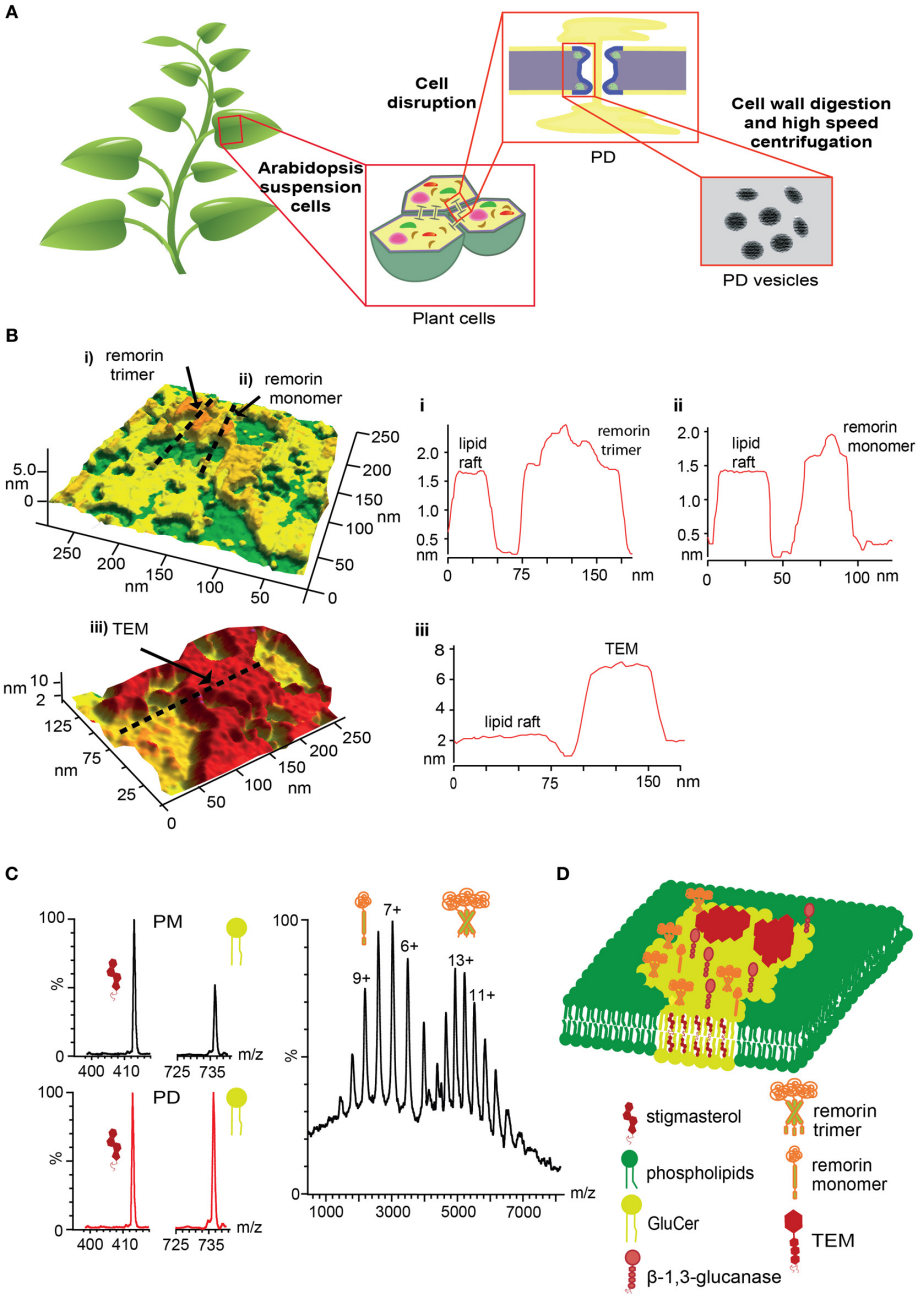
**Combined AFM and MS methodology to characterize the PD protein and lipid structure. (A)** Scheme of the purification of PD vesicles from *A. thaliana* suspension cells. **(B)** AFM imaging simulations of lipid rafts, TEMs and remorin in PD membrane. Upper panel, AFM imaging of PM (green) and lipid rafts (yellow) where remorin (arrows) monomer and trimers can be localized above lipid raft domains. Lower panel, AFM imaging of lipid rafts and TEMs (red) is graphed. Right panels show a selection of cross section analyses **(i–iii)** for lipid and protein areas indicated as dashed lines in left panels. **(C)** Mass spectra simulations of stigmasterol and GluCer lipids from PM and PD are shown in black and red lines respectively (left panel). Mass spectra simulations of intact remorin showing monomeric and trimeric stoichiometries (right panel). **(D)** PD membrane model based on AFM and MS results.

It has been widely accepted that complementary structural techniques including AFM and MS are needed to understand and predict the behavior of intact membrane proteins (Barrera and Edwardson, 2008; Barrera and Robinson, 2011). Additionally, AFM has proved to represent an excellent and unique choice to visualize the dynamics of lipid rafts (Henderson et al., 2004). In this perspective article we propose a combination of these methods to get novel insights into the protein and lipid composition of the PD, in particular to determine the presence of lipid rafts, membrane protein stoichiometry and lipidomics. Considering that PD is a dynamic structure that responds to environmental stimuli, probably by changing its protein composition (Maule, 2008), we propose to evaluate *in vitro* purified PD vesicles under different physiological conditions. As stated in Figure 1A, Fernandez-Calvino et al., 2011 reported a methodology to purify PD vesicles, which are derived from membrane fractions without significant cell wall according to electron microscopy imaging. Further analysis by immunoblot of the samples confirmed the presence of PD proteins like PDLP1, and the absence of proteins associated to ER (BiP), Golgi (Membrine 11) and chloroplast (thylakoid P16).

Working with purified PD vesicles under physiological conditions (Figure 1A), AFM imaging could be applied to identify lipid rafts and TEMs based on topological parameters. Expected heights above plasma membrane for lipid rafts and TEMs are 1–4 nm (Cai et al., 2012; Orsini et al., 2012) and 5–6 nm (Brisson et al., 1983; Taylor and Robertson, 1984; Walz et al., 1995; Min et al., 2002, 2003), respectively. A putative AFM imaging of the PD vesicles is shown in Figure 1B. PM areas correspond to the minimal height of the vesicles (green color) which should be composed mostly by phospholipids. Lipid rafts correspond to flat domains of 1.5–2 nm above plasma membrane (yellow color). TEMs correspond to flat domains of 5–6 nm above plasma membrane (red color). Using MßCD on PD vesicles we could trigger a reorganization of the lipid rafts but not affecting TEMs (Claas et al., 2001; Giocondi et al., 2004) which would allow us to differentiate both membrane domains. Membrane proteins should be also observed in some areas of the lipid rafts and a variety of hypotheses could be tested. For example, remorin has been proposed as lipid raft molecular marker and has been found in the PD (Table 1). AFM imaging of this protein, based on its molecular weight, would induce a particle height less than 1 nm above the lipid raft (Figure 1B, upper panel). Remorin can also form homotrimers *in vitro* where each subunit is anchored to the membrane via C-terminal tails (Perraki et al., 2012). Therefore, assuming remorin (arrows, Figure 1B) can present different stoichiometries in the lipid rafts, AFM imaging could detect monomers (Figure 1B, ii) as well as trimers (Figure 1B, i) over the lipid raft flat surface. This is based only on approximate changes on molecular area or volume between both structures. As other proteins can be present in this membrane domain, another strategy is needed to corroborate the presence of this protein, which is force spectroscopy, using AFM tip functionalized (Dufrêne et al., 2013) with a specific anti-remorin antibody (Lefebvre et al., 2010). This technique consists of chemical modifications of the AFM tip to make a covalent bond between the tip surface and a protein/biomolecule (probe). In consequence this probe can interact specifically with the sample. In this case, at the single molecule event, a significant binding affinity on the remorin/anti-remorin antibody complex would trigger an increase of the interaction force that subsequently can be transformed in dissociation constant (Le et al., 2011) for the remorin-antibody binding reaction. This figure can then be compared with traditional experiments to analyse binding such as isothermal calorimetry or surface plasmon resonance, usually used for much larger amount of sample. As stated previously, GPI-anchored proteins have been also proposed as lipid raft molecular markers in mammalian and plant cells (Sangiorgio et al., 2004; Mongrand et al., 2010) and therefore ß- 1,3-glucanase, localized in the PD (Table 1), is another alternative to study protein localization in the lipid rafts via AFM. Despite technical advances, visualization of PDs in living cells by AFM is currently precluded by the PD location, in the cell-to-cell physical communication that is not accessible to the AFM tip.

To characterize the lipidomics of PD vesicles we could use Liquid Chromatography-MS/MS. Based on Mongrand et al. (2004), it is expected that GluCer would be distributed in DRMs rather than in PM with a peak at 736.6 m/z. On the other hand, stigmasterol (peak at 412.7 m/z) would be in similar proportions in PM and lipid rafts. Figure 1C shows mass spectra of both lipid species. Hence MS of PM lipids would show a larger stigmasterol/GluCer intensity ratio (black spectra in Figure 1C) compared to a MS of PD lipids (red spectra in Figure 1C) from plant cell samples. As recently demonstrated, MS has been used to determine the stoichiometry of intact membrane protein complexes as well as to identify post-translational modifications and small molecules bound to membrane proteins. This can be done by removing the protective micelle environment via collision-induced dissociation with neutral gas molecules inside mass spectrometer that results in the releasing of intact membrane proteins (Barrera and Robinson, 2011; Barrera et al., 2013). Using MS on solubilized PD vesicles, it would be possible to determine the stoichiometry of remorin and test whether or not form monomers and/or trimers (Figure 1C), and therefore corroborate the data obtained by AFM imaging (see above). The measurement can be very accurate within Da resolution. In addition, the same sample could be digested by trypsin in proteomic experiments to verify possible protein modifications.

In our experimental design we have proposed to work under different physiological conditions to analyse variations on PD constitution and conformation in response to environmental stimuli. It is reported that changes on PD permeability by pathogen infection have been observed after increasing callose deposition on the cell wall near to the PD neck, which reduces channel diameter (Zavaliev et al., 2011). In addition, the cellular redox state regulates PD permeability. Mutations in mitochondrial RNA helicase, *ise1*, (Stonebloom et al., 2009), and thioredoxin type m3, *gat1*, (Benitez-Alfonso and Jackson, 2009), induce an increase of reactive oxygen species (ROS) in plant cells. However, both mutants have opposite effects on the PD permeability, while *ise1* increases permeability, *gat1* decreases it. More recently, H_2_O_2_ treatments display a byphasic effect on PD permeability, where 0.6 mM and 6 mM H_2_O_2_ produce a two-fold increment and total abolition, respectively (Rutschow et al., 2011). These results confirm that redox state controls the PD permeability and makes it an important candidate to modulate the lipid and protein abundance in the PD. Therefore, we propose to incubate cultured plant cells with low (0.6 mM) and high (6 mM) H_2_O_2_ concentrations prior to PD vesicles purification. There is evidence showing that the plasmodesmal aperture is regulated by callose deposition at the neck region (Simpson et al., 2009; Zavaliev et al., 2011). Therefore, an increase in permeability induced by treatment with low concentrations of H_2_O_2_ could be mediated in part by the increase in the abundance of β-1, 3 - glucanase (protein that degrades callose) and/or a decrease in the abundance of plasmodesmal callose binding protein (PDCB1). It has been shown that PDCB1 overexpression augmented callose accumulation resulting in a reduction of green fluorescent protein (GFP) diffusion. Therefore, there is an association between PDCB-mediated callose deposition and plant cell-to-cell communication (Simpson et al., 2009). An opposite effect would be observed after high H_2_O_2_ concentrations. Interestingly, protein clustering in lipid rafts depends on cholesterol presence (Simons and Toomre, 2000). Moreover oxygenated derivatives of cholesterol (oxysterols) can be generated by ROS (Terao, 2014) and trigger a dynamic redistribution of lipids from lipid rafts (Bacia et al., 2005). For example, if stigmasterol is similarly modified, an oxysterol signal would appear in the MS lipidomics analysis and probably would affect the size and dynamics of the lipid raft imaged by AFM. These evidence suggest that cellular redox states may change PD permeability thorough lipid and protein modifications, which can be studied by traditional lipidomics and proteomics in MS experiments.

These proposed experiments would allow us to postulate a structural PD membrane model (Figure 1D) where specific lipid and protein components are responsible for the mechanisms underlying biomolecule transport.

In this perspective article, we have discussed the potential use of complementary state-of-the-art AFM and MS to characterize the PD lipid and protein structure from native conditions. We envisage that novel studies in the near future combining this with plant genomics could lead to an integrative view on the PD role for cell-to-cell communication throughout plant development.

### Conflict of interest statement

The authors declare that the research was conducted in the absence of any commercial or financial relationships that could be construed as a potential conflict of interest.
